# Terahertz imaging for early screening of diabetic foot syndrome: A proof of concept

**DOI:** 10.1038/srep42124

**Published:** 2017-02-06

**Authors:** G. G. Hernandez-Cardoso, S. C. Rojas-Landeros, M. Alfaro-Gomez, A. I. Hernandez-Serrano, I. Salas-Gutierrez, E. Lemus-Bedolla, A. R. Castillo-Guzman, H. L. Lopez-Lemus, E. Castro-Camus

**Affiliations:** 1 Centro de Investigaciones en Optica A.C., Loma del Bosque 115, Lomas del Campestre, Leon, Guanajuato 37150, Mexico; 2 Departamento de Matematicas y Fisica, Centro de Ciencias Basicas, Universidad Autonoma de Aguascalientes, Av. Universidad #940, Ciudad Universitaria, C.P. 20131, Aguascalientes, AGS, Mexico; 3 Hospital Angeles Leon, Av. Cerro Gordo 311, Lomas del Campestre, 37150 Leon, Guanajuato, Mexico; 4 Instituto de Seguridad y Servicios Sociales de los Trabajadores del Estado, Hospital Regional Leon, Calle Pradera 1101, Azteca, 37520 Leon, GTO, Mexico

## Abstract

Most people with diabetes suffer some deterioration of the feet. Diabetic foot syndrome causes ulceration in about 15% of cases and such deterioration leads to amputation in about 2.5% of diabetic patients, diminishing their quality of life and generating extraordinary costs for patients and public health systems. Currently, there is no objective method for the detection of diabetic foot syndrome in its early stages. We propose terahertz imaging as a method for the evaluation of such deterioration. This screening method could aid the prevention and medical treatment of this condition in the future.

With a current world prevalence close to 8% and growing, one of the most important health conditions affecting people globally is Diabetes Mellitus (DM). DM is characterized by abnormal levels of blood glucose^1^, which in turn leads to long term deterioration of many organs and tissues in the body^2^. The prevalence of DM is rising alarmingly and figures in certain countries of the world have gone above 20%^3^.

The so called “diabetic foot”, or “diabetic foot syndrome”, is a common consequence of DM. The feet of diabetic people present, in many cases, a combination of microvascular and neurological deterioration which results in poor irrigation and loss of sensitivity in their feet^4^. The combination of these two conditions causes dehydration of the skin, which in turn becomes more fragile, together with a compromised capacity to notice small lesions. This results in an increased risk of patients to develop severe ulcers, which in many cases require partial or total amputation of the limb; this is the most common cause of non-traumatic amputations^5^.

At present, the only strict definition of diabetic foot we were able to find is that of “a foot affected by ulceration that is associated with neuropathy and/or peripheral arterial disease of the lower limb in a patient with diabetes”^6^. Unfortunately, this definition does not allow much room for early diagnosis, and is the consequence of the lack of an objective and quantitative screening method that allows preventive actions; at the same time such lack of an objective screening method has also made the assessment of preventive treatments difficult. The most used podiatric evaluation of diabetic patients before they present ulcers is a subjective test known as the monofilament that assesses the neurological deterioration^4^. This test consists in poking the feet of a blinded patient with a flexible tip and asking the patient to report when he/she feels the pressure, when the patient fails to report a number of pokes, the outcome of the test is considered to be positive^7^. Another indirect test is the brakial-ankle index, which uses doppler ultrasound to compare the blood flow in arteries in the arm and ankle, which is an indicator of vascular deterioration^8^. Other image studies are also used, but they are normally suitable only for relatively advanced stages^9^,^10^.

The number of applications of terahertz radiation in the biological^11^,^12^,^13^ and medical^14^,^15^ fields has exploded in recent years, owing to its unprecedented sensitivity to the water content of tissues. In addition, this technique is non-invasive and entirely harmless to living organisms, given that terahertz photons have around three orders of magnitude less energy than that needed for ionization, and that most terahertz time-domain spectroscopy sources operate in the sub-1 *μ*W power regime. Terahertz has been used to detect skin^16^, breast^17^, colon^18^,^19^, and other types of cancer^20^, as well as a tool to determine the severity of skin burns, which is not always easy to diagnose clinically^21^,^22^.

In this article, we propose terahertz reflection imaging as a potential tool for the detection of deterioration in the feet of diabetic patients. Terahertz radiation is a highly sensitive, non-invasive, non-contact probe of the water content of materials. We assume that dehydration of the skin of the feet of diabetics owing to peripheral vascular disease is a central element of their deterioration process, and therefore, terahertz imaging can be an excellent diagnostic tool. We present an appropriate imaging device for such purpose, and some evidence of terahertz imaging as a potential and effective early screening method. We want to emphasize the fact that this article presents preliminary evidence, but it is not the report of a formal clinical trail. Yet, the results are encouraging and provide key elements that will allow the design of a clinical trail in the future. Furthermore, this is, to our knowledge, the largest sample of living humans ever reported to be imaged with terahertz for a medical application. As part of this report, we also provide an analytical expression for the dielectric function of dehydrated human skin and a model for the dielectric function of any hydration level.

## Setup Design

**Figure 1:**
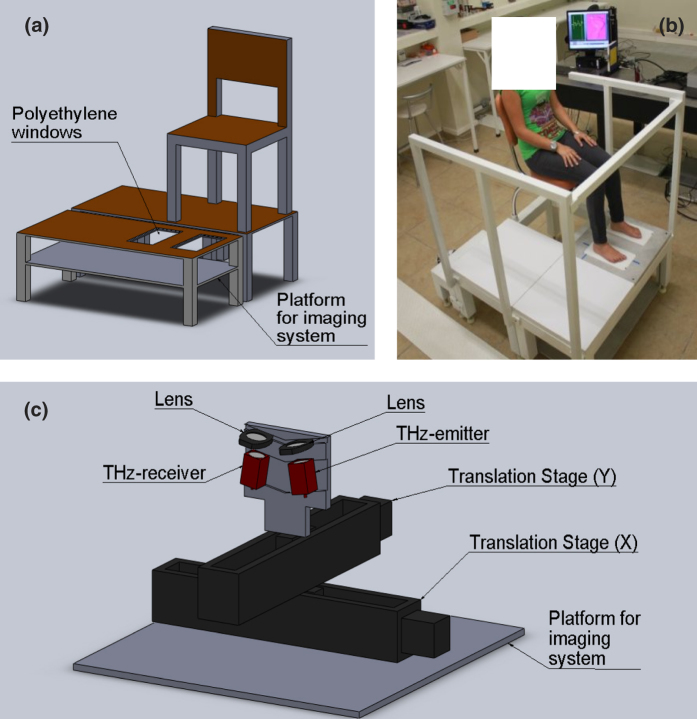
(**a**) Terahertz setup design. (**b**) Photograph of the setup once assembled while being tested. (**c**) Schematic representation of the raster-scanning imaging array which is placed on the platform under the windows as indicated in (**a**).

Most of the foot ulcers experienced by diabetic patients occur on the sole, in either the greater toe, the metatarsal area (the region where the toes meet the rest of the foot) and the heel area; therefore an image of the entire foot sole is required. Although some terahertz cameras are available^23^, they are still relatively scarce and they do not provide spectral information. As we will show in the following sections, spectral information is required in order to extract the water content of the tissue at each point; therefore, a full spectroscopic imaging system that produces terahertz images of the foot sole is required. We built a platform, shown in Fig. 1, that consists of an elevated surface where two high-density polyethylene windows, which are transparent at terahertz frequencies, are used to place the patients’ feet. A chair is also provided in order to maintain the patient comfortably in position and avoid motion of the feet during the image acquisition. The space under the patient sitting area is used to place a terahertz time-domain spectrometer and a raster scanning system, which is used to produce the image from underneath. The terahertz time-domain system will be described in detail in the methods section.

## Results

A total of 33 volunteers were recruited among students and employees from the Centro de Investigaciones en Optica, which we designated as the “control group”; although no standard screening test for DM was applied, none of them were diagnosed diabetics. Additionally, 38 diagnosed diabetic individuals from the “*Hospital Regional Leon*” of the “*Instituto de Seguridad y Servicios Sociales de los Trabajadores del Estado*” were recruited and designated as the “diabetic group”. The right foot of all subjects was imaged, with the exception of that of one of the diabetic patients, who had already undergone a right-foot amputation; in that case, the left foot was imaged. Of all the images acquired, only 21 of the control group and 12 from the diabetic group are included in this report; the others were discarded because the subject moved during the image acquisition, making the image inappropriate for analysis.

**Figure 2:**
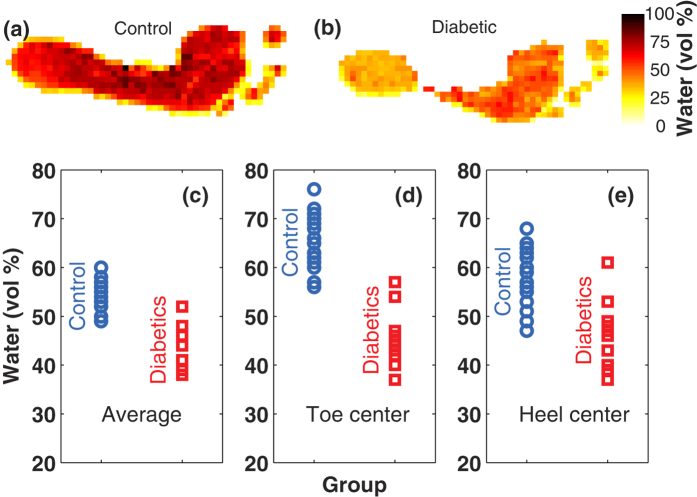
(**a**) Terahertz image of a typical member of the control group. (**b**) Terahertz image of a typical member of the diabetic group. Volumetric fraction of water for control group members and diabetics (**c**) averaged over the foot sole, (**d**) at the center of the greater toe and (**e**) at the center of the heel. Each point represents a subject.

The raw data of each image were then processed with the algorithm described in the Methods section in order to obtain the water content of the skin at each point of the image. A typical control subject and a typical diabetic subject image are shown on Fig. 2a,b, respectively. The two images show a remarkable difference, with the water content of the control subject being significantly larger than that of the diabetic subject. In order to summarize the results of all the images, the average water content, the water content at the center of the greater toe and the water content at the center of the heel are shown in Fig. 2c–e, respectively. Notice that the two groups can clearly be distinguished in all three plots, being the hydration of diabetic subjects lower than that of control subjects.

**Figure 3:**
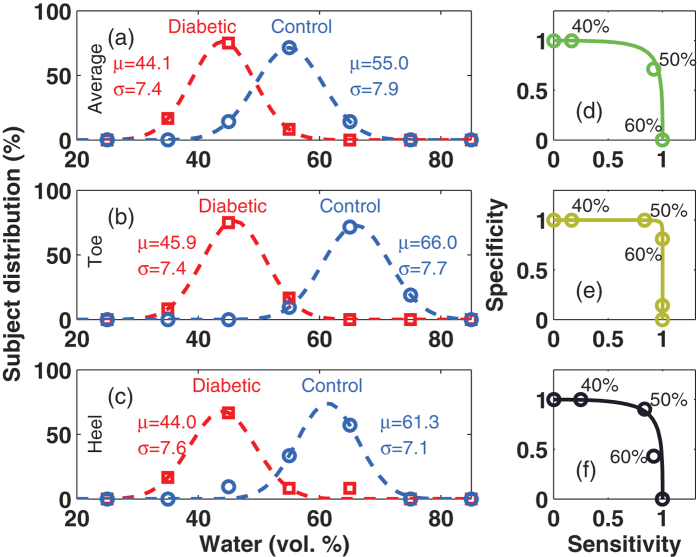
The statistical distribution of both the diabetic (squares) and control (circles) groups is shown in the plots for the average water content across the sole (**a**), the greater toe (**b**) and the heel (**c**). The dashed lines are gaussian fits to the data, and the mean and standard deviation of the fits are provided in each plot. Panels (d–f) are the *Receiver Operating Characteristic* curves which are used to define the threshold values for diagnostic tests for the average, toe and heel measurements.

The tests for the two groups have the statistical distributions shown in Fig. 3. The points on each plot were calculated with the numbers extracted from the images and are shown in Fig. 2c–e. The dashed lines are gaussian fits from which the mean and standard deviation could be calculated; these numbers are shown in the plot. The plot shows a clear separation of the diabetic and control populations with all three measurements (average, toe and heel).

In order to quantify the potential of a diagnostic test, a clear definition of a positive and negative result has to be defined. Given that there is no previous knowledge on the normality ranges of hydration, we will define thresholds for each of the three measurements that optimize the sensitivity and specificity of these tests. The sensitivity is a fractional measure of how good a diagnostic test is to give a positive result for people that actually suffer the condition, and is defined as


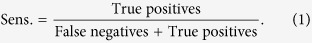


On the contrary, the specificity of a test is a fractional measure of its capacity to detect the absence of the condition in a person not suffering it, and is defined as


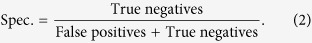


The optimization of the thresholds for a diagnostic test can be guided by the use of the *Receiver Operating Characteristic* curves (shown in Fig. 3d–f); the region where the “elbow” of the curve appears is the region where the threshold should be defined. By defining a threshold of 50% for the average water content, we obtain the distribution of subjects shown in Table 1. From this table, an estimation of sensitivity and specificificity of the test can be calculated assuming that all diabetics will have certain degree of deterioration in their feet, unlike those in the control group. In this case, the sensitivity turns out to be 0.92, and the specificity 0.86. Analogous tables are made for the greater toe and heel measurements using thresholds of 57% and 50% respectively (Tables 2 and 3).

Based on Tables 2 and 3, the resulting sensitivity and specificity of the toe test are 1.0 and 0.90, and the ones for the heel test are 0.83 and 0.90 respectively. It is worth emphasizing that these values are just indicative, and a careful discussion of their meaning and their limitations is provided in the following section.

## Discussion

The images reported in the previous section, together with the quantitative analysis done on the numbers extracted from them, demonstrate that there are significant differences between the hydration of the feet of subjects in the control and the diabetic groups. Furthermore, the assumption that most, if not all, the diabetic group members show a degree of deterioration in their feet makes it reasonable to attribute the differences observed in the terahertz measurements to the deterioration of the feet of diabetics. The estimations of a sensitivity in the range 0.83 to 1.0 and a specificity between 0.86 and 0.90 are rather encouraging. Such numbers will be extremely helpful in the planning of a formal clinical trail for this technique as a screening method for early stages of diabetic foot syndrome.

While indicative and encouraging, the sensitivity and specificity values reported here are far from being acceptable for clinicians. Our study was initially proposed as a technical test of the equipment and, therefore, many formal requirements of a clinical trail were not met in the acquisition of the data presented here. For instance, the size of our sample is clearly insufficient, the subjects in the control group did not undergo a reference test, actually a *golden standard* for “early” diabetic foot syndrome was not even defined, and finally there is a significant difference in the average age of the control and diabetic groups, which could result in statistical bias of our observations, and therefore in an overestimation of the sensitivity and specificity. Terahertz imaging is a non-invasive method that subjects patients to no risk and has the advantage of probing the hydration state of the skin directly, unlike the monofilament or the Doppler tests, which probe the deterioration of the nerves or the vasculature, respectively. However, terahertz imaging remains a relatively expensive technique.

In conclusion, we proposed terahertz reflection imaging as a potential screening method for early stages of feet deterioration in diabetic patients. We also designed and implemented an appropriate equipment for the acquisition of feet sole images. In addition, we acquired images of 12 diabetics and 21 non-diabetics, which gave us a very optimistic indication of the potential of the technique. Based on these results, we are now planning a clinical trail of the screening method, which should be completed in the coming months or years. Yet, we would like to emphasize that, provided that our observations are confirmed in a wider and more rigorous clinical trail, this method could radically change the approach to the medical care of the feet of patients with diabetes, as well as to the development of new treatments for this condition.

## Methods

### Terahertz time domain spectroscopy

The terahertz system we used was based on an Er:fiber laser which produced 90 fs pulses at a repetition rate of 100 MHz, at an average power of 120 mW and central wavelength of 1550 nm. The pulses were split into two parts; the first one was fiber coupled and sent to an InGaAs photoconductive emitter in order to produce terahertz transients, while the second part of the pulse was sent to a computer controlled delay-line, and subsequently, fiber coupled and sent onto an InGaAs photoconductive detector. Further details of the operation of photoconductive devices can be found in ref. 24.

The terahertz system could be configured in transmission geometry with the emitter and detector facing each other with four lenses forming a single optical axis that collects the terahertz radiation from the emitter, focuses it onto the sample, collects the transmission and focuses it again onto the detector. This geometry was used for the measurement of the dielectric properties of dehydrated skin samples. For the generation of the feet images, a reflection geometry was adopted. In that case, the emitter and detector were placed on a CNC-machined mount that kept them facing two optical axes 25° from each other. The mount was designed to position two high-density polyethylene lenses in such a way that their focal point matched the exact position where the two optical axes crossed, therefore forming a 12.5° “pitch-catch” geometry (see Figs 1a and 6). The mount was placed on a pair of orthogonal translation stages which were in turn positioned on a plate parallel to the polyethylene windows that held the patients’ feet as mentioned earlier. The raw image data consists of a collection of terahertz waveforms taken across a square mesh of 22 × 54 points spaced by 5 mm for each patient.

### Determination of the dielectric function of dehydrated human skin

In order to obtain a hydration image from the reflected terahertz radiation, we require to build a relationship between the hydration level of skin and its terahertz optical properties. Hydrated skin can be seen as the combination of dehydrated skin and water. Given that the complex dielectric properties of water are well known^25^, the remaining component to be characterized is dehydrated skin. The pathology laboratory of the “Hospital Regional Leon” of the “Instituto de Seguridad y Servicios Sociales de los Trabajadores del Estado” provided 8 samples of healthy skin obtained from the remaining tissue of breast biopsies. Approximately the first millimeter of the skin (mainly epidermis) was dissected from subcutaneous soft tissue with an scalpel. The skin (epidermis) was formalin fixed (10% diluted formaldehyde with distilled water) and subsequently dehydrated with 96% ethanol and absolute ethanol (99.5%). The samples where then rehydrated, flattened by applying mild pressure with a mechanical press, and dehydrated in absolute ethanol again in order to obtain a flat sample.

**Figure 4:**
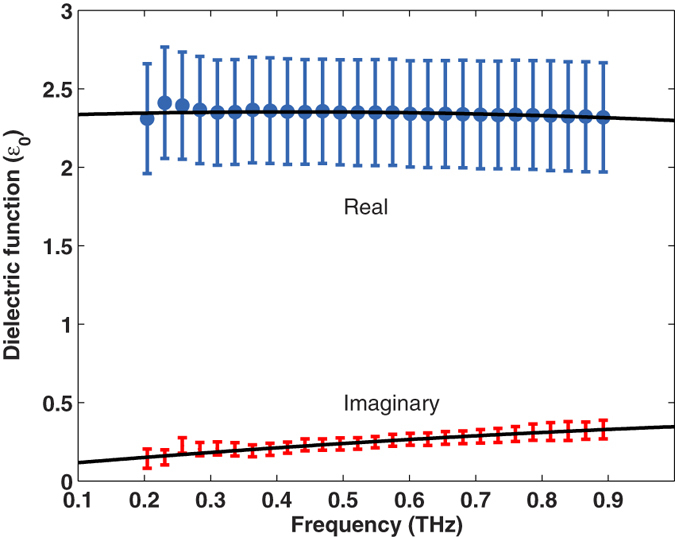
Complex dielectric function of dehydrated human skin, the continuous curves are second order polynomials fit to the data which are valid between 0.2 THz and 0.9 THz.

A terahertz time-domain spectrometer was used in order to measure the transmittance of the human skin samples. The data were subsequently processed as described in ref. 26 in order to obtain their complex dielectric function. The complex refractive index of the 8 different samples from different biopsies were averaged and the curves obtained are shown in Fig. 4. The error bars shown represent the standard deviation of the 8 measurements. Second order polynomials were fit to the both the real and imaginary parts of the average dielectric function in the range from 0.2 THz to 0.9 THz, where we considered the measurements are valid; outside this range, the signal to noise ratio dropped below +5 dB (electric field). From the measurements, we are able to estimate that the dielectric function of human skin can be approximated by


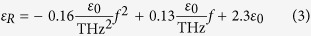


and


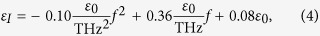


where *f* is the frequency in THz. These expressions are valid from 0.2 THz to 0.9 THz and are shown in Fig. 4 as continuous lines.

### Effective medium theory

**Figure 5:**
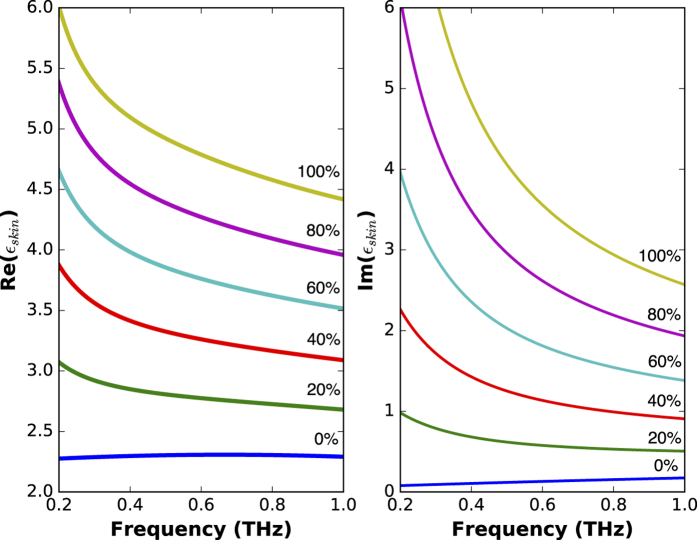
Complex dielectric function of human skin for various volumetric fractions of water.

In order to obtain an analytic expression that relates the terahertz reflection to the water content of the skin, we used the Landau-Lifshitz-Looyenga effective medium model^27^. In this model, the dielectric function of a mixture of two substances, dehydrated skin and water in our case, can be calculated provided that the dielectric function of the two substances is known, as well as the volumetric fraction occupied by each. The dielectric function of the hydrated skin is given by


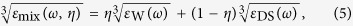


where *η* is the volumetric fraction of water, *ε*_W_ is the well known dielectric function of water^25^, and *ε*_DS_ is the dielectric function of the dehydrated skin given by the polynomials introduced in the previous section. With this expression, the dielectric function of any hydration state of the human skin can be calculated, as shown in Fig. 5.

### Transfer function for reflection imaging and inverse problem solution

**Figure 6:**
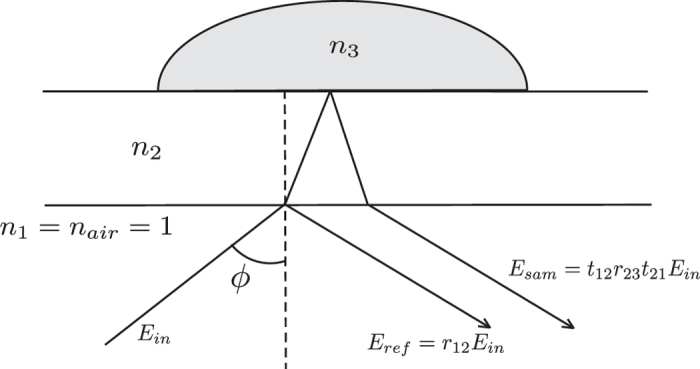
Ray schematic of the terahertz radiation path.

The final step in order to obtain the hydration images is to correlate the terahertz time-domain data taken for each pixel of the image measured with the local water content. For this, we built a transfer function that can be calculated from the experimental data for each pixel, and that can also be modeled theoretically as a function of the water volumetric fraction *η*. If we assume an incoming terahertz pulse, whose waveform is given by *E*_in_ (Fig. 6), that reaches the bottom of the polyethylene windows, the part of the pulse reflected at the first surface would be given by


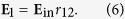


The transmitted part will propagate through the window and will reach the window-foot interface, in which the pulse will be partly reflected; that reflection will propagate again through the window, and part of it will be transmitted at the interface, which will be given by


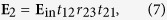


in these expressions, *t*_*ij*_ and *r*_*ij*_ are the Fresnel transmission and reflection coefficients at the interface between *i*-th and *j*-th material. Therefore, if we measure the reflected waveform as a function of time, it will consist of two pulses separated in time. The spectral amplitude of these two pulses is given by


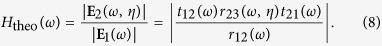


The Fresnel coefficients can be calculated from the refractive index of the materials involved. Notice that all those refractive indices are constant and well known, except for the refractive index of the foot skin, which depends on the water content of the skin, as explained in the previous section.

As for the experimental measurements, the thickness of the windows used (10 mm) is large enough so that the pulses are well separated in time. If we numerically Fourier transform the two pulses separately and keep only the amplitude information, we can build the experimental transfer function


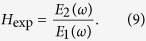


Yet, factors such as the slight misalignment of the two beams, as shown in Fig. 6, are factors hard to theoretically account for; therefore, it is a common practice to obtain an empirical calibration factor^28^ by measuring the experimental transfer function of a position where there is no foot, i.e. where the two reflections are associated to the air-polyethylene and polyethylene-air interfaces. Given that these two materials have well known dielectric properties, it is possible to calculate the factor


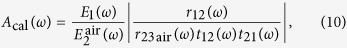


needed in order to match the theoretical expression and the experimental measurement. This calibration factor is subsequently incorporated to the expression in Eq. 8, resulting in a redefinition of


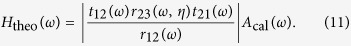


All the Fresnel coefficients present in this expression depend on the refractive index of polyethylene and air only, except for *r*_23_(*ω, η*), which is given by


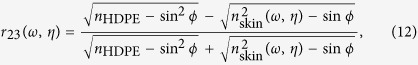


where *ϕ* = 12.5° is the incidence/reflection angle and 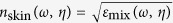 from Eq. 5. In order to obtain the volumetric fraction of water at each point of the image, a least square fitting algorithm was used in order to minimize the difference between the experimental and the theoretical transfer function by varying the parameter *η*.

### Imaging of human subject feet

The subjects were requested not to apply any moisturizing creams or similar products for at least 24 hours before the image acquisition. Their shoes were removed about 5 minutes before they were placed on the polyethylene windows of our imaging system. Then, the image was acquired, taking around 25 minutes in total each. The surface of the windows and the area around them was cleaned with isopropanol between measurements.

This study was approved by the Research Ethics Committee of “Hospital Regional ISSSTE Leon” on the 18/Sep/2014 and the study was conducted in accordance with all relevant guidelines and regulations in Mexico. All patients signed an informed consent form in order to take part in it.

## Additional Information

**How to cite this article:** Hernandez-Cardoso, G. G. *et al*. Terahertz imaging for early screening of diabetic foot syndrome: A proof of concept. *Sci. Rep.* **7**, 42124; doi: 10.1038/srep42124 (2017).

**Publisher's note:** Springer Nature remains neutral with regard to jurisdictional claims in published maps and institutional affiliations.

## Figures and Tables

**Table 1:** Distribution of diabetic and control subjects as a function of foot sole average water content.

	Diabetic	Control
Average W% < 50%	11	3
Average W% > 50%	1	18

**Table 2:** Distribution of diabetic and control subjects as a function of greater toe center water content.

	Diabetic	Control
Toe W% < 57%	12	2
Toe W% > 57%	0	19

**Table 3:** Distribution of diabetic and control subjects as a function of heel center water content.

	Diabetic	Control
Heel W% < 50%	10	2
Heel W% > 50%	2	19
